# Collectanea Medica, Consisting of Anecdotes, Facts, Extracts, Illustrations, Queries, Suggestions, &c.

**Published:** 1818-08

**Authors:** 


### COLLECTANEA MEDICA,

CONSISTING OF

ANECDOTES, FACTS, EXTRACTS, ILLUSTRATIONS, QUERIES, SUGGESTIONS, &c.

RELATING TO THE

*History or the Art of Medicine, and the Auxiliary Sciences.*

Quicquid agunt medici,
nostri farrago libelli.

*On the Sounds produced by Flame in Tubes, &c.* by M. FARADAY, Chemical Assistant in the Royal Institution. [From the Journal of Science and the Arts, No. X.]

THERE is an experiment usually made in illustration of the properties of hydrogen gas, which was first described by Dr. Higgins, in the year 1777,|| and in which tones are produced by

---

|| Nicholson's Journal, Vol. 1, page 130.

burning a jet of hydrogen within a glass jar or tube. These tones vary with the diameter, the thickness, the length, and the substance of the tube or jar; and also with changes in the jet. They have frequently attracted attention, and some attempts to explain their origin have been made.

After Dr. Higgins, Brugnatelli in Italy, and Mr. Pictet at Geneva, described the experiment, and the effects produced by varying the position and other circumstances of the jet and tube ; and M. de la Rive read a paper at Geneva (published in the Journal de Physique, LV. 165.) in which he accounted for the phenomenon by the alternate expansion and contraction of aqueous vapour. That they are not owing to aqueous vapour, will be evident from some experiments to be described. I have no doubt they are caused by vibrations, similar to those described by M. de la Rive; but the vibrations are produced in a different manner, and may result from the action of any flame.

I was induced to make a few experiments on this subject, in consequence of the request of Mr. J. Stodart, that it should be introduced at one of the evening meetings of the Members and Friends of the Royal Institution ; and was soon satisfied that no correct explanation had been given. That the sounds were not owing to any action of aqueous vapour, was shewn by heating the whole tube above 212° ; and still more evidently by an experiment, in which I succeeded in producing them from a jet of *carbonic oxide*. That they do not originate in vibrations of the tube, caused by the current of air passing through it, was shewn by using cracked glass tubes, tubes wrapped up in a cloth, and, I have obtained very fine sounds by using a tube formed at the moment by rolling up half a sheet of cartridge paper, and keeping it in form by grasping it in the hand. The sounds have been accounted for, as well as their supposed peculiarity of production by hydrogen, by the supposition of a rapid current of air through the tube; but that this is not essential, is shewn by using tubes closed at one end, and bell glasses, as described by Mr. Higgins, in his first experiment.

I was surprised to find, on my first trials with other gases, that I could produce those sounds from them which had been supposed to be generated exclusively by hydrogen ; and this, with the insufficiency of the explanations that had yet been given, induced me to search after the cause of an effect, which appeared to be produced generally by all flame.

In examining attentively the appearance of a flame when introduced into a tube, it will commonly be found, that, on coming within its aperture, a current of air is established through the tube, which compresses the flame into a much smaller space : it is slightly lengthened, but its diameter is considerably diminished: on being introduced a little further, and as the tube becomes warm, this effect is increased, and the flame is gradually compressed a little above its commencement at the orifice of the jet, more than at any other part; a very faint sound begins to be heard, and as it increases, vibrations may be perceived in the flame, which are most evident in the upper part, but frequently also perceptible in the lower and smaller portion: these increase with the sound, which at last becomes very loud, and if the flame be further introduced into the tube, it is generally blown out. Such are the general appearances with hydrogen. If a jet of olefiant, or coal gas, both of which I have ascertained may be used successfully, be substituted; then, in addition to those appearances, it will be perceived that, as the bright flame of the gas enters the tube, its splendour is diminished, and it burns with less light.

By substituting other gases and inflammable vapours for hydrogen, and using other vessels than tubes, I was enabled so to magnify the effects, as to perceive more distinctly what took place in the flame at these times; and soon concluded, that the sound was nothing more than the report of a continued explosion.

Sir H. Davy has explained the nature of flame perfectly; and has shewn that it is always a combination of the elements of explosive atmospheres. In continued flame, as of a jet of gas, the combination takes place successively, and without noise, as the explosive mixture is made. In what is properly called an explosion, the combination takes place at once throughout a considerable quantity of mixture, and sound results from the mechanical forces thus suddenly brought into action; and a roaring flame presents something of the appearance of both. If a strong flame be blown on by the mouth, a pair of bellows, the draught of a chimney, or other means, the air and the gaseous inflammable matter are made to mix in explosive proportions in considerable quantities at once, and these being fired by the contiguous flame, combine at once throughout their whole extent, and produce sound: the effect is rapidly repeated in various parts of the flame as long as the air is mixed thus forcibly with it, and a repetition of noise is produced, which constitutes the roar.

Now this, I believe, to be exactly analogous to that which takes place in what have been called the singing tubes; but in them the explosions are generally more minute and more rapid. By placing the flame in the tube, a strong current of air is determined up it, which envelopes the flame on every side. The current is stronger in the axis of the tube than in any other part, in consequence of the friction at the sides and the position of the flame in the middle; and just at the entrance of the tube an additional effect of the same kind is produced, by the edge obstructing the air which passes near it; the air is therefore propelled on to the flame, and mingling with the inflammable matter existing there, forms portions of exploding mixtures, which are fired by the contiguous burning parts, and produce sound, in the manner already described, with a roaring flame; only, the impelled current being more uniform, and the detonations taking place more rapidly and regularly, and in smaller quantities, the sound becomes continuous and musical, and is rendered still more so by the effect of the tube in forming an echo.

That the roaring flame gives sound in consequence of explosion, can hardly be doubted; and the progress from a roar to a musical tone, is easily shewn in the following manner: take a lamp with a common cotton wick, and trim it with ether or alcohol; light it, and hold a tube over the flame (that which I have used is a thin tube of glass about an inch in diameter, and near thirty inches long;) in a few seconds after introducing the flame, the draught will be sufficiently strong to blow it out, but if the current be obstructed by applying the fingers round the lamp at the bottom of the tube, combustion will go on, though irregularly; then, by a little management in admitting the air on one side or the other, and in greater or lesser quantity, it may be impelled on to the flame in various degrees, so as to produce a rough roaring sound, or one more continued and uniform, of a higher note, and more musical; and these may be made to pass into each other at pleasure: then, by substituting a stream of etherial vapour for the wick, which may be easily done from a small flask through a tube, the tones may be brought out more and more clearly, until they exactly resemble those of hydrogen.

A similar experiment may be made with coal gas: light a small Argand burner with a low flame, and bring a glass tube, which is very little larger than the diameter of the flame, down upon it so as nearly to include it: the current of air will be impelled on the external part of the flame, it will remove the limit of combustion a little way up from the burner, that part of the flame will vibrate rapidly, burning with continued explosions, and an irregular tone will be obtained. Remove the burner, and fix on a long slender pipe to the gas tube, so as to afford a candle flame that may be introduced into the tube; light it, and introduce it about five or six inches, and a clear musical tone will be obtained.

During the experiments that were made in consequence of this view of the subject, many appearances occurred which might be added to the above account, to support the opinion that the vibration of the flame, in consequence of rapid successive explosions, is the cause of the sound; but they are neglected, because they are supposed unnecessary.

If the explanation given be true, then the only requisite to the production of these sounds is the successive sudden inflammation of portions of gaseous explosive mixtures. These mixtures are most easily made by propelling a stream of air on to a stream of inflammable gaseous matter: but it is also possible to make them in other ways, and the same phenomenon may be produced in a different manner.

That the tube is not essentially necessary, is shown by making it swell out into a cylinder of three or four inches diameter, except above and below; or part of it may be extended into a globe. I took two air jars that were open above, but with contracted apertures; one of these was inverted over an inflamed jet of hydrogen, so as to form a lamp or bell glass about it: there was no effect of sound, because the downward currents from above interfered with the stream of air issuing up from beneath, and made it irregular; but placing the second receiver on the first, applying them edge to edge; so as to preserve the current of air upwards from disturbing forces, the sounds were immediately produced: and, lastly, I succeeded in obtaining the tones by the draught of a common chimney; for, by attaching a large inverted air jar to the end of a funnel pipe that came from the flue, closing the other lower opening into it, and introducing an inflamed jet of hydrogen within the lower contracted orifice of the glass, the sounds were produced.

That the same sounds may be obtained by means different to those above described, though depending on the same cause, is shewn by some experiments made by Sir H. Davy, in his first researches on the miners' safety lamp. Small wire-gauze safety lamps being introduced into air jars filled with explosive atmospheres, the gases burnt on the inside of the cylinder, and produced sounds similar to those obtained from a jet of flame in a tube.

Having thus endeavoured to account for the phenomenon of sounds produced by jets of flame in tubes and other vessels, I shall notice shortly the combustible bodies I have tried. Carbonic oxide, olefiant gas, light hydrocarbonate, coal gas, sulphuretted hydrogen, and arsenuretted hydrogen were burned at the end of a long narrow brass tube rising up from a transferring jar placed under pressure in a pneumatic trough. Ether was burned from the end of a tube fixed in a flask containing a small quantity which was heated; but a better method, and one I afterwards adopted, is to pour a little ether into a bladder, and then force common air in; so much ether rises in vapour as to prevent the mixture being detonating, and it may be pressed out, and burnt at the end of a tube. All these were very successful. Alcohol was more difficult to manage from being less volatile; but it succeeded when raised in vapour from a flask and burnt at a tube. In trials made with a wax taper, no distinct tone could be produced; but when the tube was made very hot, so as to assist the current through it, something like the commencement of a sound was heard at the moment the taper was blown out by the current.

Hydrogen is by far the best substance by which to produce these tones; and its superiority depends upon the low temperature at which it inflames, the intense heat it produces in combustion, and the small quantity of oxygen that a given bulk of it requires. It is in consequence less easily extinguished by the current than other gases, the current formed is more powerful and rapid, and an explosive mixture is sooner made. With gases producing little heat by combustion, and therefore occasioning but a feeble current, the effect is increased by first heating the tube at a fire, and when not heated previously, the tone is perceived to improve as the tube becomes hot from the flame playing in it.

Some variations of the form of the vessel inclosing the flame, and the material used, have been mentioned. Globes from seven to two inches in diameter, with short necks, give very low tones: bottles, Florence flasks, and phials have always succeeded: air jars, from four inches diameter to a very small size, may be used. I constructed some angular tubes of long narrow slips of glass and wood, placing three or four together, so as to form a triangular or square tube, tying them round with packthread, and easily obtained tones from hydrogen by means of them; and it is evident that variations of the channel, the use of which is to form and direct the current of air, may be made without end.

---

### *An Account of the New Alkali lately discovered in Sweden.*

[From the same.]

The discovery made by M. Arfwedson of a new alkali, and announced in the last number of that Journal, has been abundantly confirmed by the researches of other chemists. Its importance has drawn the attention of the whole chemical world, and much new information relative to its nature and its sources, has since been added, which we shall endeavour to comprise in the following account of this body.

Lithia (the name given to the new alkali) was first found in the petalite, a mineral from the mine of Utoen in Sweden. It may be obtained very readily by fusing the mineral with potash, dissolving the whole in muriatic acid, evaporating to dryness, and digesting in alcohol: the muriate of lithia being very soluble in that fluid, is taken up, whilst the other salts remain, and, by a second evaporation and solution, is obtained perfectly pure.

The muriate is itself a very characteristic salt of the alkali. It may easily be decomposed by carbonate of silver, and the carbonate treated with lime yields pure lithia.

The exact quantity of lithia in the petalite is doubtful, but it cannot contain much more than five per cent. A more abundant source has however been found in the Triphane or Spodumene, which according to M. Arfwedson, who also first pointed out in it the existence of lithia, contains eight per cent. of the new alkali. The same chemist has likewise ascertained its existence in another mineral from Utoen, which is called crystallised lepidolite, in the proportion of four per cent.

The pure alkali is very soluble in water, has a very acrid caustic taste, like the other fixed alkalies, and acts powerfully on blue vegetable colours. When heated on platinum, it acts on it. It has a strong affinity for acids, and a very high neutralising power, even surpassing that of magnesia. Its solution precipitates the earthy and metallic salts, nearly as solutions of the other alkalies do.

Placed in the voltaic circuit, Sir H. Davy shewed that it was decomposed with the same phenomena as the other alkalies. A portion of its carbonate being fused in a platinum capsule, the platinum was rendered positive and a negative wire brought to the upper surface. The alkali decomposed with bright scintillations, and the reduced metal being separated, afterwards burnt. The small particles which remained a few moments before they were reconverted into alkali, and allowed a short examination, were of a white colour, and very similar to sodium. A globule of quicksilver made negative, and brought into contact with the alkaline salt, soon became an amalgam of lithium, and had gained the power of acting on water, and evolving hydrogen, an alkaline solution resulting.

The chloride of lithium obtained by evaporating the muriate to dryness, and fusing it, is a white semi-transparent body, analagous in its appearance to the chlorides of potash and soda, but very different from them in its general properties. It is extremely deliquescent, whereas they are not so: in this respect it almost equals muriate of lime. Its solution crystallises with great difficulty, but by evaporation affords minute needle-form crystals. It is very soluble in alcohol, but the chlorides of potash and soda very little so. Its solution, or the moist salt, has the property of tinging the flame of alcohol of a fine red, somewhat like strontian, but the other alkaline muriates have not this power. It fuses below a red heat, and when heated powerfully in the open air, it gradually loses chlorine, absorbs oxygen, and becomes strongly alkaline.

All its salts are very fusible, but in some cases a singular degree of insolubility belongs to them. The nitrate is a very soluble salt, deliquescent, and capable of crystallising in rhomboids. It has a very aigre taste: heated it readily fuses, and is then decomposed with the same phenomena as nitre.

The sulphate of lithia is a salt which crystallises readily in small rectangular prisms; they are perfectly white, and possess much lustre; have a saline taste, very different to potash or soda; are more soluble in water than sulphate of potash; perhaps not so soluble as sulphate of soda: the crystals contain no water; they fuse and become very liquid below a red heat; their solution does not precipitate the muriate of platina, nor is it precipitated by tartaric acid. M. Vauquelin gives an experiment on its constitution, the result of which is as follows:*

| | |
|---|---|
| Sulphuric acid - - | 69.18 |
| Lithia - - - | 30.82 |
| | 100.00 |

The sub-carbonate of lithia is but little soluble in water, and effloresces in the air. It may even be precipitated from its sulphate by adding a strong solution of carbonate of potash to it. It is readily fusible, and when fused, requires repeated additions of water with boiling to dissolve it again. Cold water dissolves about one one-hundredth part of its weight of this salt, and the solution

* There is an error in the calculations in M. Vauquelin's paper. the given constituents of 100 parts, if added together, making 101. The proportions deduced from the experiment, are correctly as above, at least to the second decimal place.

acts powerfully on vegetable colours and effervesces with acids. According to Vauquelin, it attracts carbonic acid very rapidly from the atmosphere.

The carbonate, heated on platinum, acts on it almost as powerfully as the fixed alkaline nitrates. It separates ammonia from its combinations, but is decomposed by lime and barytes, and rendered caustic.

The solution of the carbonate precipitates the muriate of lime, the sulphates of magnesia and alumina, and the salts of copper, silver, and iron, just as the other alkaline carbonates do; but it does not precipitate the muriate of platinum, as is the case with the sub-carbonate of potash.

The tartrate of lithia is an efflorescent salt; the acetate one that, on being evaporated, takes the state and consistence of gum or syrup.

The sulphuret formed by M. Vauquelin, appeared in all respects similar to the fixed alkaline sulphurets; except in the larger proportion of sulphur thrown down by acids.

With respect to the proportions of the elements of the alkali, they do not appear exactly determined. Mr. Arfwedson states, that lithia contains 43.9 per cent. of oxygen. M. Vauquelin concludes that it contains 43.5 per cent.; but in consequence of the error mentioned in the preceding note, the last number is not correct, and if deduced from that experiment, the oxygen would be 44.84 per cent.

---

### Mr. Brande *on Meteoric Stones.*

[From the same.]

Iron is the principal metallic ingredient in those lapideous masses, which in different countries have fallen upon our globe, and which have been termed meteoric stones. Though we really know nothing of the source or origin of these bodies, it has been ascertained, upon the most satisfactory and indisputable evidence, that they are not of terrestrial formation, and consequently, since men began to think and reason correctly, their visits to our planet have awakened much speculation, and some experimental research.

In the first place, it deverves to be remarked, that we have very distinct evidence of the falling of stony bodies from the atmosphere in various countries and at very remote periods. For, to say nothing of the fabulous trash which encumbers the annals of ancient Rome, or the extended catalogue of wonders flowing from the lively imagination of Oriental writers, such events are recorded in holy writ, and have been set down by the most accredited of the early historians; and although philosophic scepticism long contended against the admission of the fact, it has in modern times received such unanswerable proofs, as to be allowed by all who have candidly considered the evidence, and is only rejected by the really ignorant, or by those who, for the sake of singularity, affect disbelief.

The first tolerably accurate narration of the fall of a meteoric stone, relates to that of Ensisheim, near Basle, upon the Rhine. The account which is deposited in the church was thus: A. D. 1492, Wednesday, 7 November, there was a loud clap of thunder, and a child saw a stone fall from heaven; it struck into a field of wheat, and did no harm, but made a hole there. The noise it made was heard at Lucerne, Villing, and other places; on the Monday King Maximilian ordered the stone to be brought to the castle, and after having conversed about it with the noblemen, said the people of Ensisheim should hang it up in their church, and his Royal Excellency strictly forbade anybody to take any thing from it. *His Excellency, however, took two pieces himself, and sent another to Duke Sigismund of Austria.* This stone weighed 255 lbs.

In 1627, 27th November, the celebrated Gassendi saw a burning stone fall on Mount Vaisir, in Provence; he found it to weigh 59 lbs.

In 1672, a stone fell near Verona, weighing 300 lbs. And Lucas, when at Larissa, 1706, describes the falling of a stone, with a loud hissing noise, and smelling of sulphur.

In September, 1753, de Lalande witnessed this extraordinary phenomenon, near Pont de Vesli. In 1768, no less than three stones fell in different parts of France. In 1790, there was a shower of stones near Agen, witnessed by M. Darcet, and several other respectable persons. And on the 18th of December, 1795, a stone fell near Major Topham's house in Yorkshire; it was seen by a ploughman and two other persons, who immediately dug it out of the hole it had buried itself in: it weighed 56 lbs.

We have various other and equally satisfactory accounts of the same kind. All concur in describing a luminous meteor moving through the air in a more or less oblique direction, attended by a hissing noise, and the fall of stony or semimetallic masses, in a state of ignition. We have however evidence of another kind, amply proving the peculiarities of these bodies: it is that, although they have fallen in very different countries, and at distant periods, when submitted to chemical analysis they all agree in component parts; the metallic particles being composed of nickel and iron; the earthy of silex and magnesia.

Large masses of native iron have been found in different parts of the world, of the history and origin of which nothing very accurate is known. Such are the great block of iron at Elbogen in Bohemia; the large mass discovered by Pallas, weighing 1600 lbs. near Krasnojark in Siberia; that found by Goldberry, in the great desert of Zahra, in Africa; probably also that mentioned by Mr. Barrow, on the banks of the great fish river in Southern Africa; and those noticed by Bruce, Bougainville, Humboldt, and others in America, of enormous magnitude, exceeding 30 tons in weight. That these should be of the same source as the other meteoric stones, seems at first to startle belief; but, when they are submitted to analysis, and the iron they contain found alloyed by nickel, it no longer seems credulous to regard them as of meteoric origin. We find nothing of the kind in the earth.

To account for these uncommon visitation of metallic and lapideous bodies, a variety of hypotheses have been suggested.

Are they merely earthly matter fused by lightning? Are they the offspring of any terrestrial volcano? These were once favourite notions; but we know of no instance in which similar bodies have in that way been produced, nor do the lavas of known volcanos in the least resemble these bodies, to say nothing of the inexplicable projectile force that would here be wanted. This is merely explaining what is puzzling, by assuming what is impossible; and the persons who have taken up this conjecture, have assumed one impossibility to account for what they conceive to be another, namely, that the stony bodies should come from any other source than our own globe.

The notion that these bodies come from the moon, though it has been laughed at as lunacy, is, when impartially considered, neither absurd nor impossible. It is quite true, that the quiet way in which they visit us is against such an origin; it seems, however, that any power which would move a body 6000 feet in a second, that is, about three times the velocity of a cannon ball, would throw it from the sphere of the moon's attraction into that of our earth. The cause of this projective force may be a volcano, and if thus impelled, the body would reach us in about two days, and enter our atmosphere with a velocity of about 25,000 feet in a second. Their ignition may be accounted for, either by supposing the heat generated by their motion in our atmosphere sufficient to ignite them; or by considering them as combustibles, ignited by the mere contact of air.

While we are considering the *possibility* of these considerations it may be remembered that in the great laboratory of the atmosphere, chemical changes may happen, attended by the *production* of iron and other metals; that at all events such a circumstance is within the range of possible occurrences; and that the meteoric bodies which thus salute the earth with stony showers, may be children of the air, created by the union of simpler forms of matter. The singular relationship between iron and nickel, and magnetism, and the uniform influence of meteoric phenomena upon the magnetic needle, should be taken into the account of these hypotheses.

